# Circular RNA cMras inhibits lung adenocarcinoma progression via modulating miR‐567/PTPRG regulatory pathway

**DOI:** 10.1111/cpr.12610

**Published:** 2019-04-22

**Authors:** Chengtao Yu, Fang Tian, Jun Liu, Minhui Su, Min Wu, Xuejun Zhu, Wang Qian

**Affiliations:** ^1^ Department of Respiratory Medicine Affiliated Hospital of Nanjing University of Chinese Medicine, Jiangsu Province Hospital of Chinese Medicine Nanjing China; ^2^ School of Biomedical Engineering Shanghai Jiao Tong University Shanghai China; ^3^ Department of Pulmonary Medicine, Shanghai Chest Hospital Shanghai Jiao Tong University Shanghai China

**Keywords:** circular RNA, cMras, lung cancer, miR‐567, PTPRG

## Abstract

**Objectives:**

Circular RNA, a type of RNA formed by a covalently closed loop, possesses sophisticated abilities of gene regulation in tumorigenesis and metastasis. However, the role of circRNAs on lung adenocarcinoma (LUAD) remains largely unknown.

**Materials and methods:**

The role of cMras was examined both in vitro and in vivo. cMras expression in LUAD tissues was determined by quantitative real‐time PCR (qRT‐PCR). Downstream targets of cMras were predicted by bioinformatics tools and confirmed by RNA immunoprecipitation assay and luciferase assay. qRT‐PCR and western blot assay were used to detect the expression of specific targets.

**Results:**

Thirty‐six paired LUAD and healthy tissues were collected and cMras resulted significantly downregulated in cancerous tissues. Its expression was negatively associated with tumour stages. cMras overexpression suppressed LUAD growth and metastasis, while endogenous cMras silencing resulted in the opposite effects. Bioinformatics analysis and experimental evidence confirmed that cMras was a sponge of miRNA‐567 and released its direct target, PTPRG. cMras overexpression decreased miR‐567 expression and subsequently increased PTPRG expression, while increased miRNA‐567 expression blocked the effects induced by cMras. Moreover, PTPRG was downregulated in LUAD and patients with low PTPRG expression exhibited significantly poor prognosis. These results suggested that cMras/miR‐567/PTPRG regulatory pathway might be associated to LUAD tumorigenesis and development.

**Conclusions:**

A novel circular RNA cMras and its functions were identified, discovering a cMras/miR‐567/PTPRG regulatory pathway in LUAD tumorigenesis and development.

## 1 INTRODUCTION

Lung cancer is a severe public health problem, representing one of the most common types of malignancy worldwide, and lung adenocarcinoma (LUAD) is its most common histological type.^1^, ^2^, ^3^ Although coding RNAs had been studied for hundreds of years to fight tumours, their therapeutic effects show a limited improvement. Thus, in order to improve the survival of patients, it is crucial to find novel molecular markers that could be beneficial in tumour early diagnosis and therapy.

Non‐coding RNAs are RNA molecules that are not translated into proteins. Abundant and functionally regulatory types of non‐coding RNAs include circular RNAs (circRNAs), microRNAs (miRNAs) and long non‐coding RNAs.^4^ circRNAs, a group of important endogenous non‐coding RNAs, mainly consist of transcripts from exons and exert important roles in the downstream gene regulation.^5^, ^6^ Unlike linear RNAs, such as mRNAs that possesses 5′ and 3′‐end at both extremities, circRNAs are circularized because free 3′‐ and 5′‐ends join together, forming a circular structure. Thus, compared to their linear counterparts, circRNAs are extraordinarily stable in vivo due to their resistance to exonuclease.^7^, ^8^, ^9^, ^10^ Recent evidences suggest that circRNAs are involved in several diseases, including tumour development. For example, circRNA has_circ_100395 inhibits lung cancer progression through the miR‐1228/TCF21 axis, while ciRS‐7 promotes oesophageal squamous cell carcinoma tumour growth and metastasis by inducing miR‐7/HOXB13 axis.^11^, ^12^

miRNAs are another group ofnon‐coding RNAs containing approximately 22 nucleotides, with a role of regulating gene expression.^13^ A recent evidence suggests that miRNAs are involved in tumorigenesis and tumour development, functioning as gene silencers at a post‐transcriptional level.^14^ In addition, transcripts can regulate each other by competing for miRNA in common, called ceRNAs (competing endogenous RNAs). Several studies reveal that circRNAs are sponges of many miRNAs, exerting the same function as ceRNAs in tumorigenesis.^15^, ^16^

In the present study, we found that cMras, a novel circular RNA Mras, was downregulated in LUAD tissues. Enhanced cMras expression inhibited cell proliferation and motility in vitro and in vivo, while its silenced expression had the opposite effects. As regard its mechanism of action, we discovered that cMras could function as a sponge of miR‐567, blocking it and releasing its target PTPRG. Our findings revealed a potential mechanism regulated by the cMras/miR‐567/PTPRG axis acting in the suppression of LUAD tumorigenesis and metastasis. Thus, cMras could represent a novel biomarker with antitumour effects, opening new perspectives in cancer research and therapy.

## 2 MATERIALS AND METHODS

### 2.1 Bioinformatics analysis

Circinteractome (https://circinteractome.nia.nih.gov/) was used to predict the potential miRNAs binding sites in the cMras and corresponding miRNAs to study the “miRNA sponge” mechanism.^17^ DIANA‐TarBase v8.0 software (http://diana.imis.athena-innovation.gr/DianaTools) was used to predict the potential miRNA target.^18^ RNA fold software (http://rna.tbi.univie.ac.at/cgi-bin/RNAWebSuite/RNAfold.cgi) was used to predict cMras advanced structure. UCSC Genome Browser Home (https://genome.ucsc.edu/) was used to display genomic cMras structure. KM Plotter (http://kmplot.com/analysis/index.php?p=service) was used to analyse the prognostic value of PTPRG.^19^

### 2.2 Tissue samples

A total of 36 pairs of LUAD and healthy tissues were obtained from patients diagnosed with lung cancer, who underwent surgery at Jiangsu Province Hospital of TCM, China. The specimens were snap‐frozen and stored at −80°C until use. All patients provided signed informed consent to the research. The Human Research Ethics Committee at this hospital approved the study. Patients' information is shown in Table 1.

**Table 1:** Clinical and pathologic characteristics of LUAD patients

	Numbers of patients	Relative expression	*P*‐value
Age (y)			
≤60	16	6.69626E‐05	0.49
>60	20	0.000155442	
Gender			
Male	19	5.07355E‐05	0.27
Female	17	0.000189192	
Tumour size (cm)			
<2.5	15	0.000168429	0.22
>2.5	21	2.57721E‐05	
Lymphatic metastasis			
Positive	17	4.00105E‐05	0.253
Negative	19	0.000184214	
Tumour stage			
T1 + T2	14	0.00027916	0.0347*
T3 + T4	22	1.2364E‐05	

* The significance of *P* < 0.05.

### 2.3 Cell culture and transfection

Human non‐small cell lung carcinoma (A549, H1975 and H1299) and normal lung epidermal cell line (HBE) were routinely cultured in RPMI Medium 1640 or MEM (C11875500BT; Life Technologies, Gaithersburg, MD, USA) supplemented with 10% foetal bovine serum (13011‐8611; Tianhang Biotechnology, China) and penicillin/streptomycin solution (15140‐122; Life Technologies) at 37°C in a 5% CO_2_ incubator. The siRNA against cMras, miR‐567 mimic and controls were designed and synthesized by Ribobio Biotechnology (Guangzhou, China). To overexpress PTPRG, the sequence of PTPRG was cloned into pcDNA3.1 vector. si‐cMras and siPTPRG sequences are listed in Supporting Information Table S1.

### 2.4 Circular RNA plasmid construction

Human cMras cloned sequence was achieved from Mras genomic DNA in A549 cells. Mras exon 2 sequence, 100 bp upstream and 100 bp downstream adjacent sequences were included. Recombinant plasmid pzw‐cMras was verified by direct sequencing. Primer sequences are listed in Supporting Information Table S1.

### 2.5 RNA extraction from cell line nuclear and cytoplasmic fractions

The extraction of the nuclear and outside cytoplasmic parts was performed using mirVana PARIS™ Kit (AM1556; Ambion, Austin, TX, USA), according to the manufacturer's protocol. Approximately 5 × 10^7 ^cells were collected, centrifuged at low speed to remove the culture medium, washed twice in pre‐cold phosphate‐buffered saline (PBS) and placed on ice. Next, they were re‐suspended in 400 μL cell fractionation buffer and incubated on ice for at least 5 minutes. Samples were centrifuged at 4°C and 500 *g* for 5 minutes, and the cytoplasmic fraction was collected. An amount of 500 μL Cell Disruption Buffer was added to the pellets, and the sample was vortexed to divide and disrupt the nuclei until the lysate was homogenous.

### 2.6 Fluorescence in situ hybridization

Fluorescence in situ hybridization (FISH) was performed to detect the presence of cMras using a Cy3‐labelled DNA probe against 5′‐ATCTTGGACGGTCTGACCTA‐3′ sequence according to the instruction of the fluorescence in situ hybridization kit (C10910; Ribobio Biotechnology). Briefly, after fixing cells in 4% paraformaldehyde, they were hybridized using the hybridization buffer using specific probes and incubated at 42°C overnight, followed by image acquisition. 18S RNA probe was used as the cytoplasm marker and U6 RNA probe was used as the nuclear marker.

### 2.7 RNase R treatment

One unit of RNase R (526413; Epicentre Technologies Corp, Madison, WI, USA) digests 1 µg of total RNA. Reaction mixtures were placed in a water bath at 37°C for 10 minutes with or without RNase R followed by phenol/chloroform and ethanol precipitation.

### 2.8 RNA extraction and quantitative real‐time PCR

Total RNA was extracted from patients' tissue samples and cell lines by Trizol Reagent (15996‐026; Invitrogen, CA, USA). Quantitative real‐time PCR (qRT‐PCR) primers were synthesized from Sangon Biotech (Shanghai, China). GAPDH or U6 was used as an internal control and the relative gene expression was calculated using the $2^{-\Delta \Delta C_{T}}$ method. Primer sequences are listed in Supporting Information Table S1.

### 2.9 Cell viability assay

Cell viability was evaluated using a CCK‐8 Kit (HY‐K0301; MedChemExpress, USA). Approximately, 1 × 10^3^ cells per well were seeded in 96‐well plates (four replicates for each group). After 1, 2, 3, 4 and 5 days incubation, 10 μL of the CCK‐8 reagent was added to each well and cells were incubated at 37°C for 1.5 hours. The optical density was read at 450 nm by the synergy 2 (Molecular Devices, Bio‐Tek, CA, USA).

### 2.10 Transwell assay

Lung cancer A549 cell line and H1299 cell line in 200 μL serum‐free media were placed into the upper transwell chamber to perform the migration assay or placed into the upper transwell chamber covered with matrigel to perform the invasion assay (8.0 μL pore size; BD Biosciences, Franklin Lakes, USA). After 20 hours, cells were incubated with 1% crystal violet for 5 minutes after fixation in 4% paraformaldehyde. Colonies were examined under the microscope by Image Scanner (GE, USA).

### 2.11 Colony formation assay

Approximately 500 cells were seeded in 6‐well plates for approximately 2 weeks. Subsequently, the colonies were incubated with 1% crystal violet for 10 minutes after fixation in 4% paraformaldehyde. Colonies were examined under the microscope by Image Scanner (GE).

### 2.12 Luciferase assay

A549 cells were seeded in 96‐well plates (1 × 10^4^ cells per well). cMras sponge sequence, PTPRG 3′UTR harbouring the putative sponge sites of miR‐567, and the corresponding site‐directed mutant seed sequence were cloned into the pmirGLO reporter vector (Promega, USA). The miR‐567 mimic was co‐transfected into the cells at the indicated concentration in the protocol (https://www.promega.com.cn/products/reporter-assays-and-transfection/reporter-vectors-and-cell-lines/pmirglo-dual-luciferase-mirna-target-expression-vector). Lysates were harvested and consecutively measured 48 hours after transfection. Primer sequences are listed in Supporting Information Table S1.

### 2.13 RNA immunoprecipitation

RNA immunoprecipitation (RIP) assay was performed using A549 and H1299 according to the protocol in MagnaRIP RNA‐Binding Protein Immunoprecipitation Kit (Millipore, Bedford, MA). cMras level was detected by qRT‐PCR. The data were used to compare firstly the input, then used to compare the cMas group with the control group.

### 2.14 Western blotting

RIPA buffer (P0013C; Beyotime, China) containing the protease inhibitor Cocktail (1 mmol/L, HY‐K0010; MedChemExpress) was used to lyse cells, and extracted total proteins were subjected to western blotting following a standard protocol. The primary antibody Rabbit polyclonal PTPRG antibody (1:1000 dilution) was purchased from Thermo (MA, USA). β‐actin (1:5000 dilution, 20536‐1‐AP) and the anti‐rabbit HRP‐linked secondary antibody (1:1000 dilution, ab50345) were purchased from proteintech (Cambridge, MA, USA).

### 2.15 Animal studies

All animal studies were performed in accordance with protocols approved by the Animal Experimentation Ethics Committee of Nanjing University of Chinese Medical Center (Nanjing, China). As regard tumour growth analysis in a xenograft model, 4‐ to 6‐week‐old immunodeficient mice were used. A total of 2 × 10^5^ cMras‐overexpressed and control cells (A549 or H1299) per mouse were subcutaneously injected into the right collar of the mice. Thirty days later, mice were sacrificed, and tumours weight was measured. For in vivo metastasis assay, cMras‐overexpressed and control A549 cells (5 × 10^5^ cells/mouse) were intravenously injected into the mice tail vein. Forty days post‐inoculation, mice were sacrificed and nodules developed in their lungs were analysed. Immunohistochemical analysis (IHC) for ki‐67 was performed as previously described.^20^ For the in vivo assay of PTPRG, 2 × 10^6^ A549 cells transfected with either the PTPRG overexpression vector or control vector were injected subcutaneously into the flank of each mouse. For further investigated the role of cMras/miR‐567/PTPRG regulatory pathway in LUAD progression in vivo, 2 × 10^6^ A549 cells transfected with control vector or cMras overexpression vector were inoculated subcutaneously, and all of the mice examined developed tumours at 10th day. About 5 nmol miR‐567 mimics (agomir) or siRNA against PTPRG (RiboBio) in 25 μL saline buffer was intratumorally injected into cMras overexpression tumours mass at multiple sites per mouse every 2 days during the next 20 days, then tumours were removed and weighed.

### 2.16 Statistical analysis

Data were expressed as mean ± standard deviation (SD). Student's *t* test was used to determine the difference between groups. At least three independent experiments were carried out for each assay. A *P *value <0.05 was considered statistically significant.

## 3 RESULTS

### 3.1 cMras characterization and downregulation in LUAD

**Figure 1:**
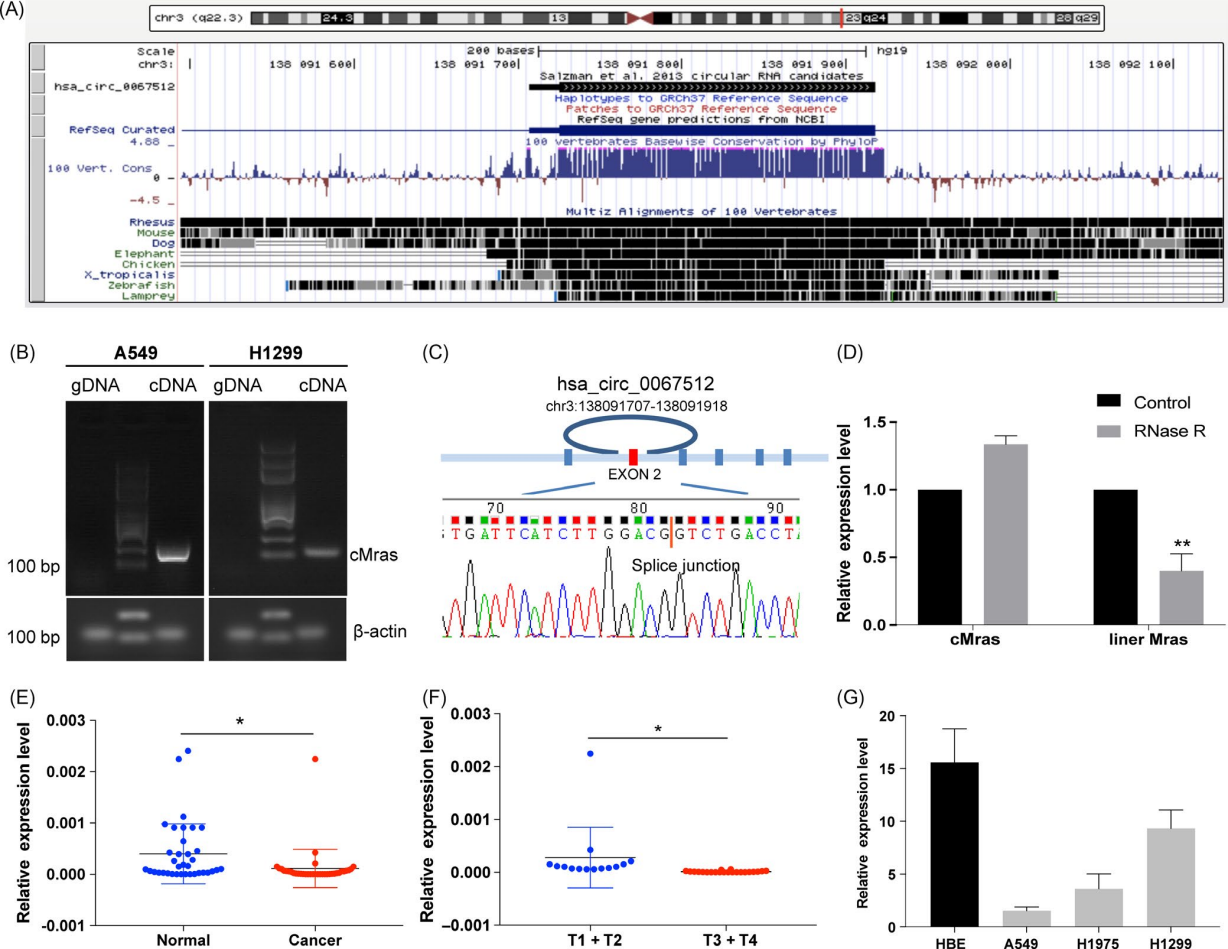
cMras characterization and downregulation in LUAD. A, cMras chromosome location and species conservation were displayed by UCSC websites. B, Gel electrophoresis of cMras PCR products amplified by divergent primers in gDNA or cDNA. C, Sanger sequencing displayed back‐spliced junction of cMras, red line pointed back‐spliced site. D, cMras and linear Mras expression were detected by qRT‐PCR after RNase R treatment. E, cMras expression was measured in 36 pairs of LUAD tissues by qRT‐PCR. F, cMras levels in different tumour stages. G, cMras expression in a panel of normal lung epithelial cell line and LUAD cell lines. Data were expressed as mean ± standard deviation of three independent experiments. *P < 0.05, **P < 0.01

Hsa_circ_0067512 (cMras) is located in the chromosome 3, 211 base pairs (bp) in length and consists of only one exon (exon 2) from the Mras genome. In addition, cMras is conserved in various species, including mouse, dog and zebrafish, suggesting its important function in biological process (Figure 1A). To verify that cMras was circular rather than products of trans‐splicing or genomic rearrangements, circRNA identification assays were performed.^7^ Divergent primers to amplify cMras were designed. Using cDNA and genomic DNA from A549 and H1299 cell line as templates, cMras was amplified from cDNA by only divergent primers, while no amplification product was observed from genomic DNA (Figure 1B). Next, the back‐spliced junction in PCR products of cMras was confirmed by Sanger sequencing (Figure 1C). Moreover, the ability of resistance to RNase R exonuclease digestion confirmed that cMras was circular in form (Figure 1D).

In order to detect cMras differential expression in LUAD and adjacent healthy tissues, its expression was evaluated in a subset of 36 pairs of LUAD samples by qRT‐PCR. The results showed that cMras was downregulated in LUAD compared with the control, suggesting its potential tumour suppressor function (Figure 1E). Moreover, cMras expression was significantly associated with tumour stages, suggesting its important role in clinical diagnosis (Figure 1F). Next, we examined cMras expression in four lung cell lines, such as three LUAD cell lines and 1 normal lung epithelial cell lines. The results showed that cMras expression was downregulated in lung cancer cell lines (Figure 1G). Taken together, these results suggested that cMras was actually circular in shape and that it was downregulated in LUAD.

### 3.2 cMras overexpression inhibited LUAD cell proliferation, migration and invasion

**Figure 2:**
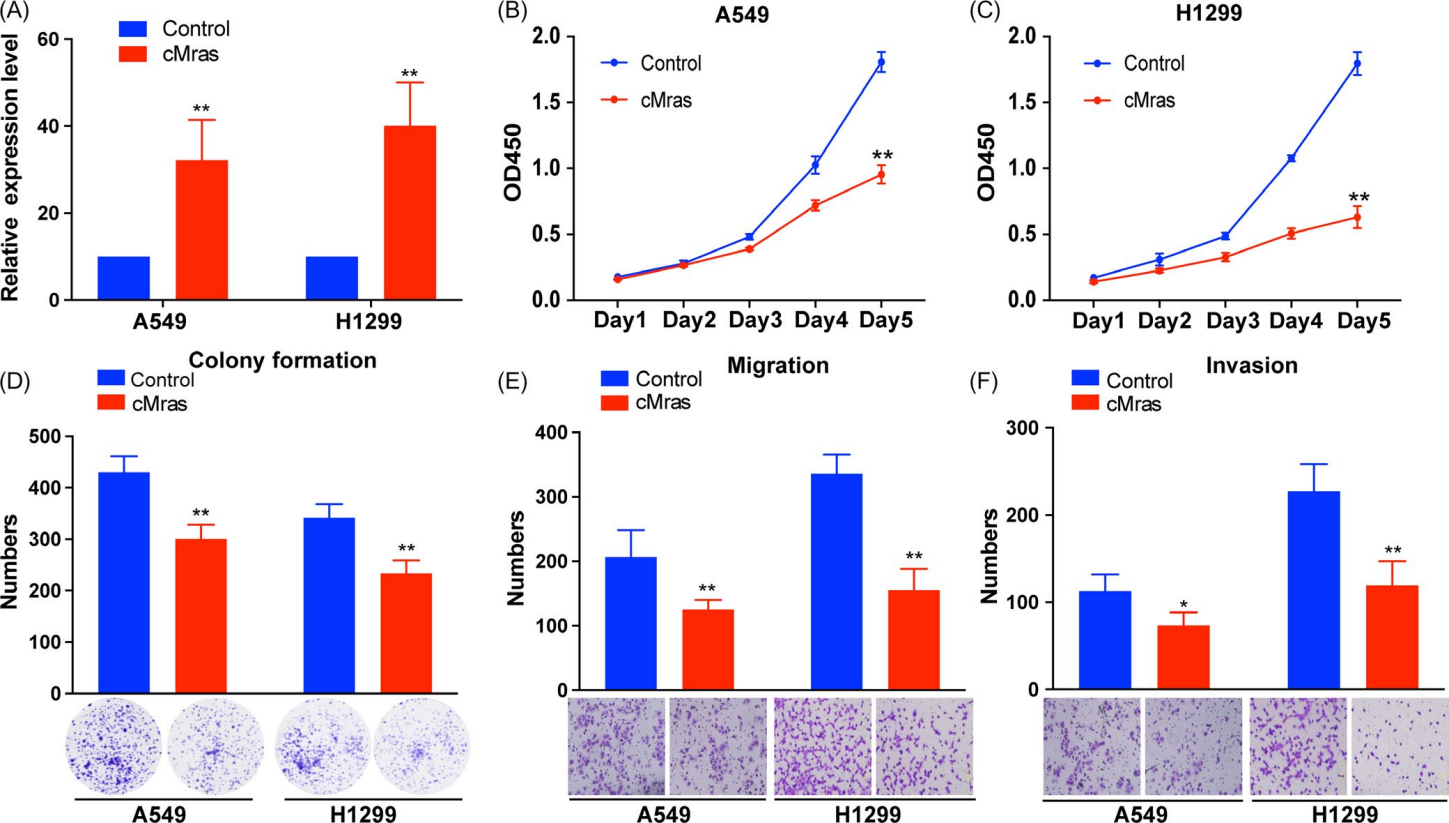
cMras overexpression inhibited LUAD cell proliferation, migration and invasion. A, cMras expression measured by qRT‐PCR after transfection with plasmid pzw‐cMras. B, C, Cell viability by CCK‐8 assay in A549 and H1299 cells transfected with empty vector (control) or pzw‐cMras. D, Colony formation assay in A549 and H1299 cells transfected with empty vector (control) or pzw‐cMras; two representative images are shown. Colony formation number was calculated by image J. E, F, Transwell assays were used to measure the migration and invasion ability of A549 and H1299 cells transfected with empty vector (control) or pzw‐cMras; two representative images are shown. Results were expressed as the number of cells per field compared with the corresponding control. Data were expressed as mean ± standard deviation of three independent experiments. *P < 0.05, **P < 0.01

Since cMras was downregulated in LUAD tissues and associated with tumour stage, the function of cMras in LUAD cancer cells was investigated. We firstly upregulated cMras expression by transfection with pzw‐cMras plasmids, and qRT‐PCR confirmed the successful cMras overexpression (Figure 2A). CCK‐8 assay revealed that the viability of A549 and H1299 was decreased in cMras overexpression group compared with that in the control group (Figure 2B,C). Consequently, colony numbers of cMras overexpressing cells were significantly less than those in the control group (Figure 2D). Furthermore, transwell migration and invasion assay indicated that the migration and invasion ability of A549 and H1299 cell line were also suppressed by cMras (Figure 2E,F). These data demonstrated that enhanced cMras expression suppressed LUAD cell proliferation, migration and invasion.

### 3.3 cMras silencing promoted LUAD cell proliferation, migration and invasion

**Figure 3:**
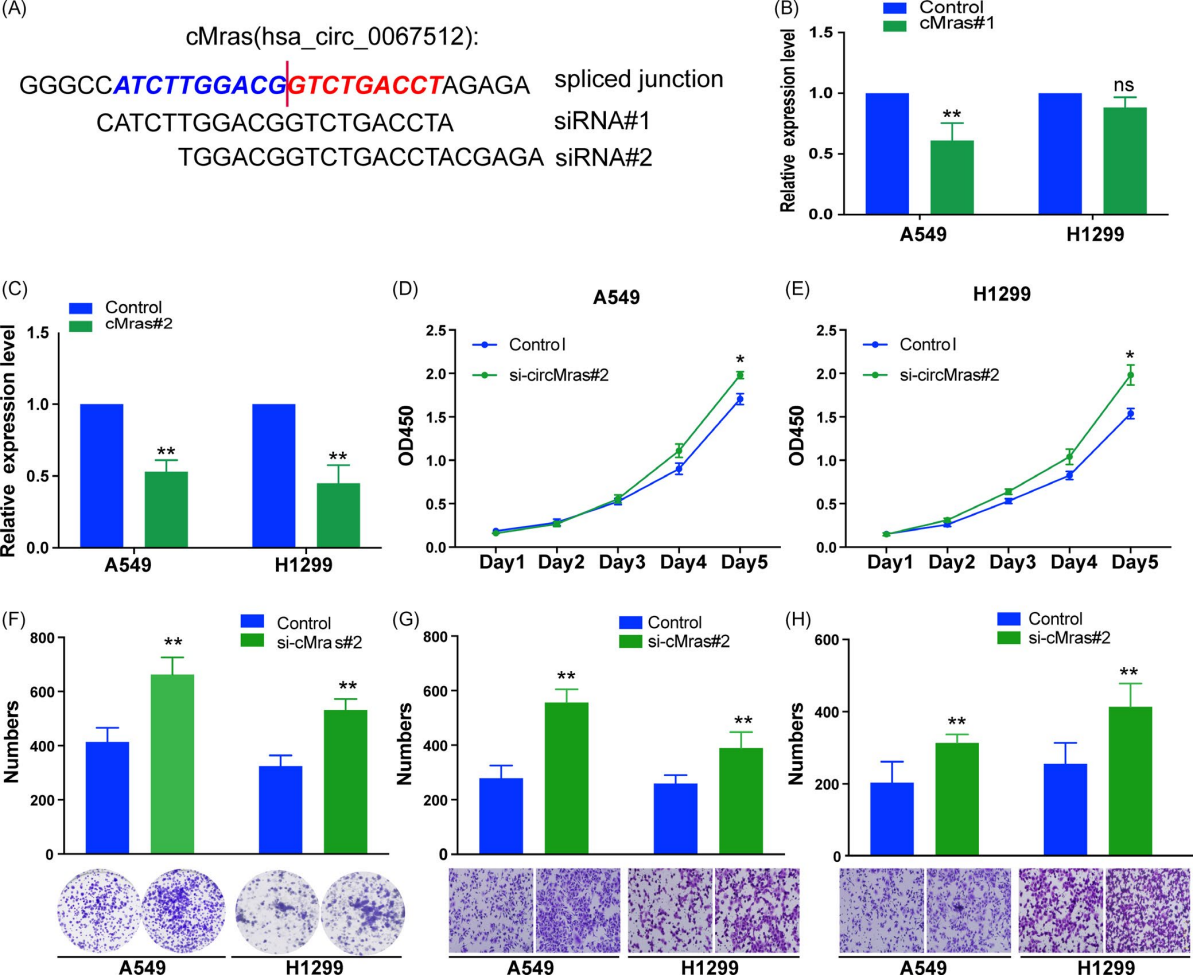
cMras silencing promoted LUAD cell proliferation, migration and invasion. A, Schematic diagram representing the designed siRNA target site. B, C, Knockdown efficiency of the two different cMras siRNAs by qRT‐PCR. D, E, Cell viability by CCK‐8 assay in A549 and H1299 cells transfected with NC (negative control) or si‐cMras. F, Colony formation assay in A549 and H1299 cells transfected with NC (negative control) or si‐cMras; two representative images are shown. Colony formation number was calculated by image J. G, H, Transwell assays were used to measure the migration and invasion ability of A549 and H1299 cells transfected with NC (negative control) or si‐cMras; Results were expressed as the number of cells per field compared with the corresponding control. Data were expressed as mean ± standard deviation of three independent experiments. *P < 0.05, **P < 0.01

Next, RNA interference was used to silence cMras in A549 and H1299 for “loss of function” investigation. Two effective siRNAs were designed to target the back‐spliced sequence of cMras (Figure 3A). The results showed that si‐cMras#2 possessed a better silencing ability both in A549 and H1299 cells (Figure 3B,C). Thus, we choose it to perform subsequent experiments. Cell proliferation assay revealed that cMras silencing significantly promoted A549 and H1299 proliferation (Figure 3D‐F). In addition, the migration and invasion were promoted by cMras silencing, as shown by the associated assays (Figure 3G,H). These results revealed that cMras silencing accelerated LUAD cell proliferation, migration and invasion.

### 3.4 cMras was a sponge of miR‐567

**Figure 4:**
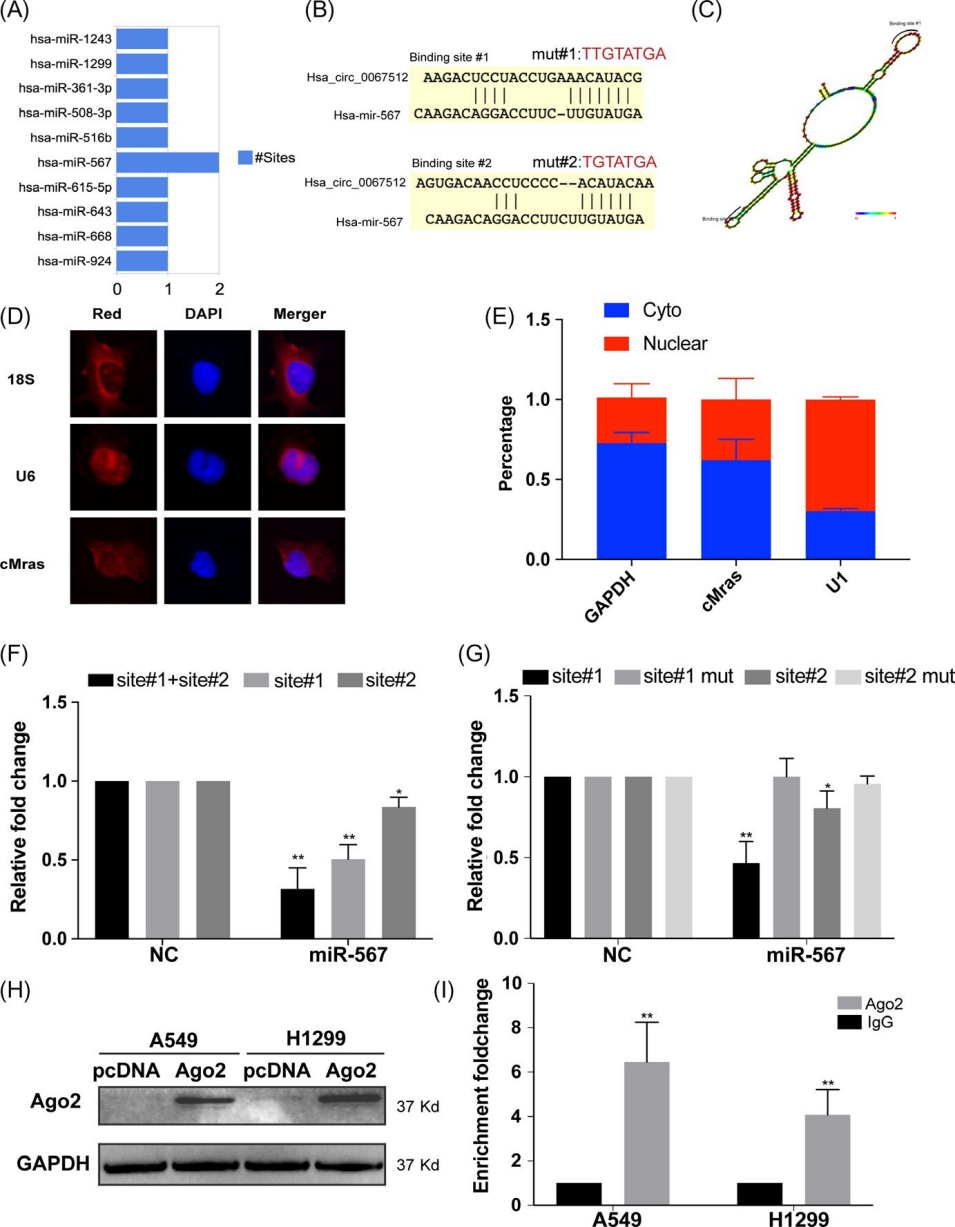
cMras was a sponge of miR‐567. A, Putative binding sites of miRNAs related to cMras. B, Wild type and mutant sequence of cMras compared with miR‐567. C, Schematic representation of cMras and miR‐567 target site. D, FISH showing the localization of cMras in A549 cells. DAPI was used to stain the nuclei; U6 was used as control; 18S RNA was the cytoplasmic control. E, U1 (nuclear control), GAPDH (cytoplasmic control) and cMras were measured by qRT‐PCR in nuclear and cytoplasmic fractions. F, Luciferase reporter assay to detect the luciferase activity in A549 cells co‐transfected with cMras binding site and miR‐567. G, Luciferase reporter assay to detect the luciferase activity in A549 cMras wild type or mutant with miR‐567. H, Ago2 protein level by western blot in A549 and H1299 cells transfected with empty pCDNA plasmid and pCDNA expressing Ago2 plasmid. I, cMras level was measured in complex of Ago2 protein in RIP assay. Data were expressed as mean ± standard deviation of three independent experiments. *P < 0.05, **P < 0.01

Recent studies revealed that circRNAs function mainly as miRNA sponges to bind functional miRNAs and then regulate gene expression.^21^ In this study, bioinformatic analysis by Circinteractome, we found that cMras shared miRNA response elements of several miRNAs, including miR‐567 with two binding sites (Figure 4A,B). Since circRNAs possess an complex structure, we predicted cMras structure by RNA fold software. Interestingly, miR‐567 binding sites were localized nearly in two loops, which was accordance with the RNA sponge theory (Figure 4C). To identify the miRNA‐binding ability of cMras, we performed FISH assay against cMras, and nuclear and cytoplasmic fraction assay to confirm that cMras was preferentially localized in the cytoplasm (Figure 4D,E). Subsequently, the luciferase assay was performed to confirm the interaction between cMras and miR‐567. The results showed that the interaction between cMras and miR‐567 in A549 decreased the luciferase activity, while the interaction between cMras mutant and miR‐567 in A549 did not influence it (Figure 4F,G). Moreover, the Ago2 RIP assay was performed to further confirm this interaction. A specific enrichment of cMras was detected in the Ago2 pulled down pellet compared with the control group (pcDNA 3.1 empty vector), supporting the evidence that cMras was a sponge of miR‐567 (Figure 4H,I).

**Figure 5:**
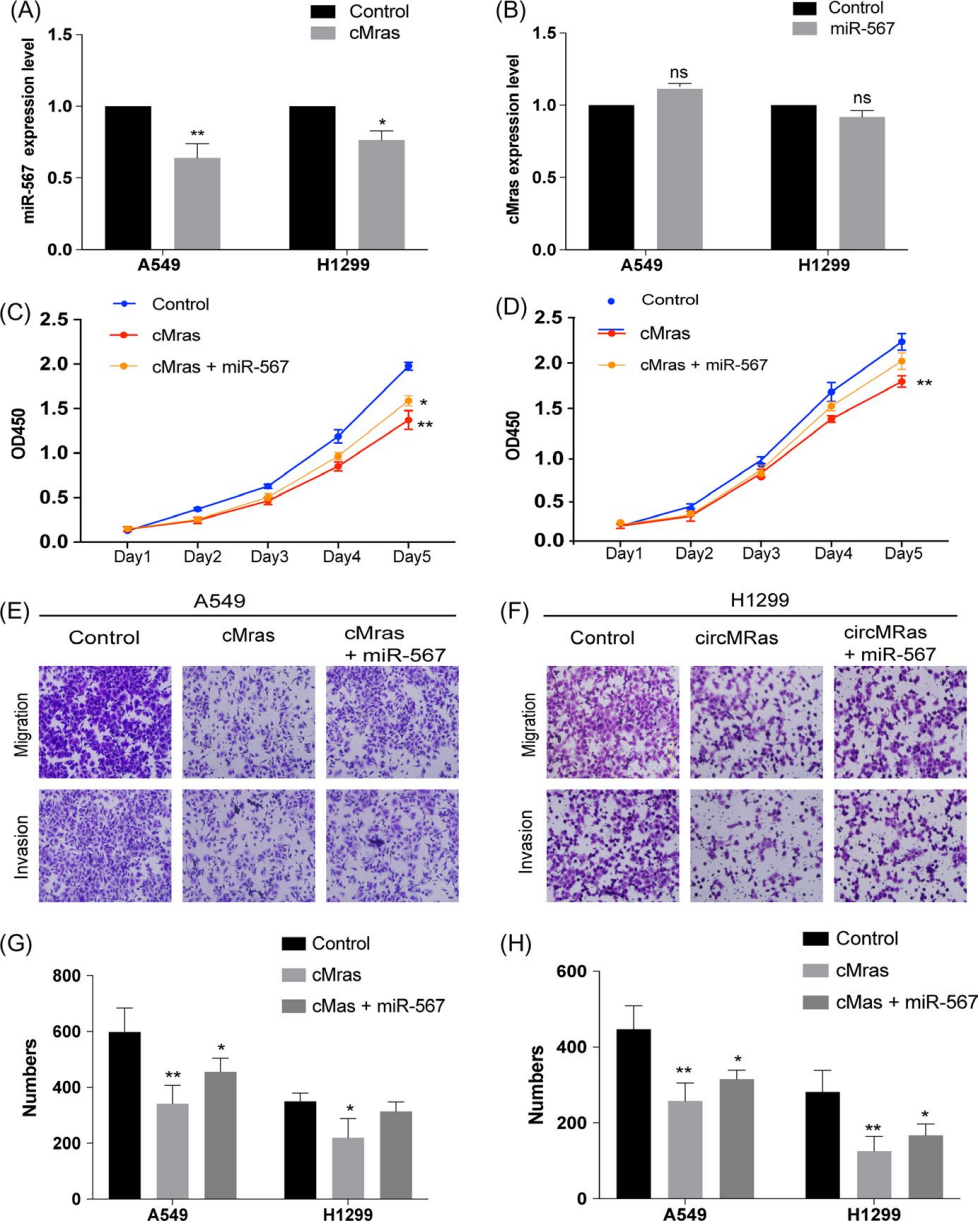
miR‐567 expression mediated the biological effects of cMras. A, miR‐567 expression after transfection with cMras in A549 and H1299 cells. B, cMras expression after transfection with miR‐567 mimic in A549 and H1299 cells. C, D, Cell proliferation measured by CCK‐8 assay in cells transfected with control, cMras and the combination of cMras and miR‐567. E, F, Transwell migration and invasion assay in cells transfected with control, cMras and the combination of cMras and miR‐567. G, H, Results were expressed as the number of cells per field compared with the corresponding control. Data were expressed as mean ± standard deviation of three independent experiments. *P < 0.05, **P < 0.01

Since we demonstrated the interaction between cMras and miR‐567, next the relationship between them in terms of mutual regulation was detected. qRT‐PCR assay showed that cMras overexpression could decrease miR‐567 expression, while miR‐567 overexpression had no effects on cMras expression, suggesting that cMras regulated miR‐567 expression (Figure 5A,B). Then, the functional aspect was further investigated. The inhibition of cell proliferation and migration induced by cMras overexpression was weakened by co‐transfection with miR‐567 mimics (Figure 5C‐F), suggesting that miR‐567 mediated cMras function in LUAD cells. Taken together, these results suggested that cMras was a sponge of miR‐567 in LUAD cells.

### 3.5 cMras inhibited the proliferation of LUAD cells by modulating miR‐567/PTPRG axis

**Figure 6:**
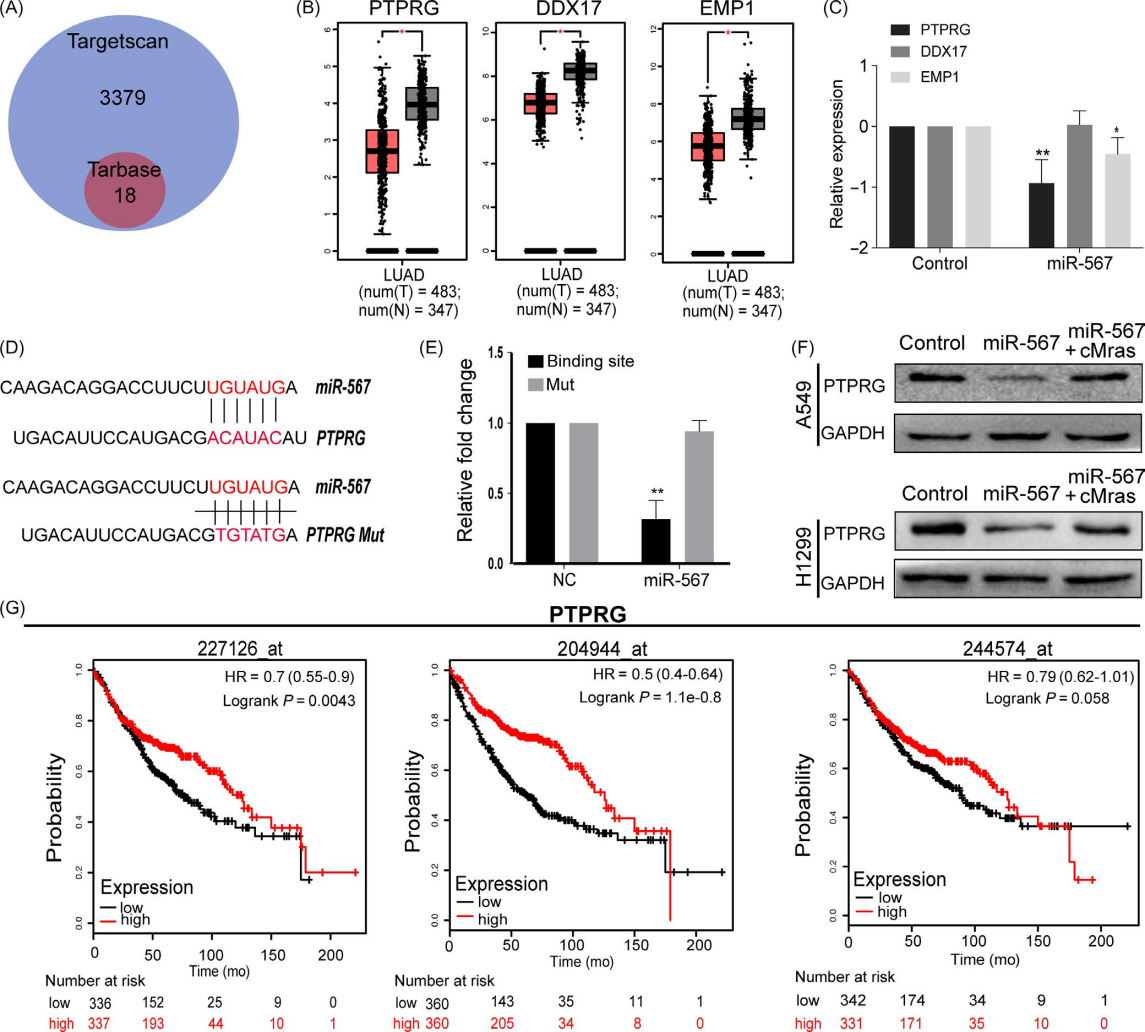
cMras inhibited the proliferation of LUAD cells by cMras/miR‐567/PTPRG axis. A, Venn diagram showing the potential targets of miR‐567 in Targetscan and Tarbase database. B, PTPRG, DDX17 and EMP1 expression in LUAD tissues by TCGA database. C, PTPRG, DDX17 and EMP1 expression detected by cMras transfection in A549 cells. D, PTPRG sequence aligned with miR‐567. E, Luciferase activity reduction observed with PTPRG wild type rather than mutant type in A549. F, PTPRG protein expression in A549 and H1299 treated with control, cMras or cMras plus miR‐567 mimic. The relative quantification was normalized by GAPDH. G, Kaplan‐Meier analysis of OS in patients with variable expression of three PTPRG probes. Data were expressed as mean ± standard deviation of three independent experiments. *P < 0.05, **P < 0.01

To find the direct targets of miR‐567, bioinformatic tools (Targetscan software) were used combined with experimental database (Tarbase v8.0). Based on the overlap targets of two tools, 18 potential targets were found (Figure 6A). Among them, only EMP1, PTPRG and DDX17 were downregulated in LUAD tissues by the analysis of TCGA database, suggesting their tumour suppression role (Figure 6B). Therefore, the investigation was further focused on these three genes and they were detected by qRT‐PCR. The results showed that EMP1 and PTPRG were both downregulated after transfection with miR‐567 mimics (Figure 6C). However, reference retrieval revealed that only PTPRG has an inhibitory role in cell proliferation and migration in lung cancer.^22^ By in vitro and in vivo assay, we confirmed that PTPRG inhibited LUAD cells proliferation and migration (Supporting Information Figure S1A‐G). Hence, PTPRG was selected for further investigation. Firstly, the 3′UTR fragments of PTPRG containing miR‐567 binding sites and their mutant fragments were cloned into the luciferase reporter vectors. A consistent reduction of luciferase activity was observed upon miR‐567 transfection in A549 cell line, while mutant fragments abolished this activity (Figure 6D,E). Functional assays suggested that PTPRG could antagonize the function of miRNA‐567(Supporting Information Figure S1I‐J). Next, whether PTPRG expression was regulated by cMras was evaluated. Western blotting showed that PTPRG protein expression was upregulated by cMras overexpression (Figure 6F). Subsequently, the analysis of its potential link with prognosis in LUAD patients revealed that patients with low PTPRG expression exhibited a significantly worse prognosis than those expressing higher levels (Figure 6G). Taken together, our evidences suggested that cMras inhibited cell proliferation and migration by modulating miR‐567/PTPRG axis in LUAD.

### 3.6 cMras affected cell proliferation in vivo

**Figure 7:**
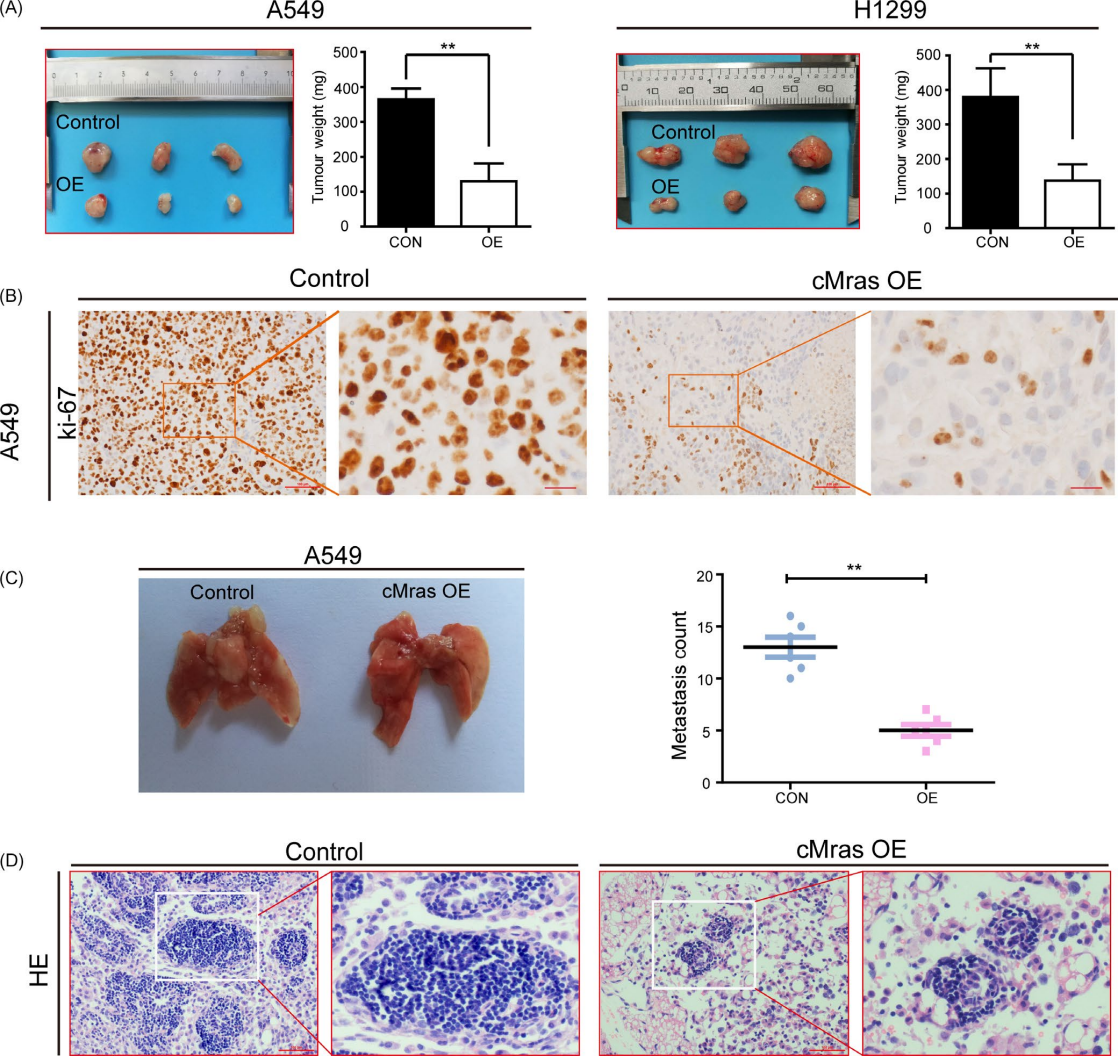
cMras affected cell proliferation in vivo. A, A549 and H1299 with control or cMras overexpression (OE) plasmid were injected in nude mice. Tumour weight was represented as mean of tumour weights ± standard deviation (SD). B, Immunohistochemical (IHC) staining of Ki‐67 in subcutaneous mice tumours. C, Lung metastasis of A549 cells after tail vein injection. Quantitative analysis of lung metastatic colonies in each group (n = 6/group). D, Representative metastatic lesions stained by H&E in the lungs of mice 4 wk after tail vein injection of the indicated cells. Scale bars: 100 μm. Data were expressed as mean ± SD of three independent experiments. *P < 0.05, **P < 0.01

To evaluate the biological function of cMras in vivo, a mouse xenograft model was established to investigate whether cMras could inhibit tumour growth. A549 or H1299 cells overexpressing cMras were subcutaneously injected into nude mice. After 35 days, the tumour size was decreased in the cMras overexpressing group when compared with the control group. Similar results were obtained for the tumour weight (Figure 7A). Moreover, tumour sections from the cMras overexpressing group exhibited weaker Ki67 staining when compared to those from the control group, suggesting that cMras overexpression inhibited tumour growth (Figure 7B). Furthermore, we established a lung metastatic model to verify the metastatic ability of cMras. The results revealed that A549‐cMras‐derived tumours possessed a smaller mass and were less numerous as compared with the control group (Figure 7C). H&E staining of lung tissues from these mice confirmed these findings (Figure 7D). Furthermore, we found that miR‐567 or siRNA against PTPRG weaken the suppressive effect of cMras overexpressing tumours (Supporting Information Figure S1H). Thus, these results demonstrated that cMras inhibited tumour growth and lung metastasis in vivo.

## 4 DISCUSSION

CircRNAs were originally considered as a by‐product of RNA transcription and their expression abundance is low. Therefore, historically, they have not been considered as crucially biological molecules.^23^ However, recent evidences showed that circRNAs can be regulatory RNAs, just like miRNA and long non‐coding RNAs, participating in several biological processes.^5^ Moreover, they are involved in tumour growth and metastasis.^24^ Lung cancer is the leading cause for cancer‐related death worldwide, and a growing number of studies suggested that circRNAs can regulate lung tumorigenesis and development.^21^ For example, X Zhu et al observed that hsa_circ_0013958 silencing suppresses the ability of proliferation and motility of cells. Besides, Qiu et al^25^ demonstrated that circPRKCI‐miR‐545/589‐E2F7 axis enhances cell proliferation and migration, and it is positively correlated with clinical feature. Similarly, higher levels of circRNA_102231 are correlated with an advanced TNM stage, lymph node metastasis and poor overall survival of LUAD patients.^26^ As a consequence of the above‐mentioned effects, circRNAs are important regulatory RNAs not only in the study of tumorigenesis and development, but also in the field of tumour therapy. In our previous study, we firstly explored circRNA has_circ_0043256 function and its involvement in the mechanism of cinnamaldehyde against lung adenocarcinoma through its action as an endogenous sponge of miR‐1252, releasing its target ITCH, and regulating Wnt/β‐catenin pathway.^20^ Thus, circRNAs were identified as important regulatory RNAs in lung cancer rather than by‐products of mRNA spliced. Despite few circRNA reports are available in cancer research, little is known regarding their role in LUAD.

In our present study, we identified a novel circRNA, cMras, by circular RNA identification assays. cMras derived from chromosome 3, exon 6 in the Mras gene locus, and named as has_circ_0067512 in circbase (http://www.circbase.org/). Then, our results showed that cMras was downregulated and negatively correlated with tumour stage in LUAD. Based on the above results, our hypothesis was that cMras might function as tumour suppressor in LUAD. Thus, the function of cMras in LUAD cell lines was analysed. The results showed that cMras inhibited tumour growth and metastasis, supporting our speculation**.**

As regard the mechanism of action, circRNAs could act as a miRNA sponge, binding RNA‐binding proteins (RBPs) and translating peptides.^27^, ^28^ Of note, circRNA acted as “miRNA sponge” regulating the downstream pathway. Our study showed that cMras had many miRNA‐binding sites, including two with miR‐567. Due to the “miRNA sponge” occurred in cytoplasm, nuclear and cytoplasm fraction assay was performed and the results showed that cMras was located mainly in the cytoplasm, suggesting the probability of a “miRNA sponge” effect. However, this result could not explain why cMras was also located in the nucleus. Thus, various functions of the cMras in LUAD cells still need to be explored beyond the “miRNAs sponges” effect.

**Figure 8:**
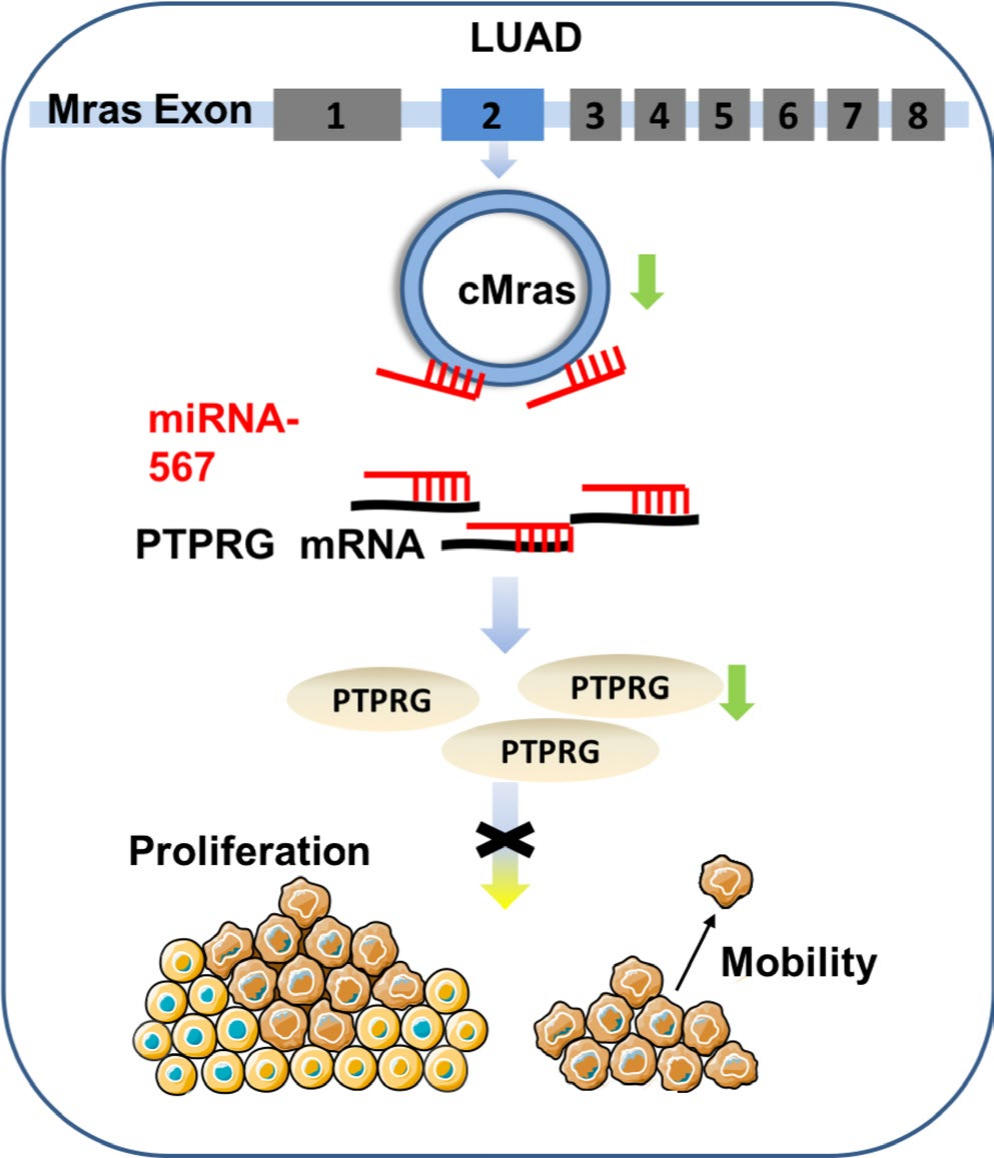
Schematic illustration of the biological role of cMras in LUAD carcinogenesis. cMras can bind to miR‐567 as a miRNA sponge, exerting its function via regulating the downstream target PTPRG; cMras knockdown can promote LUAD cell proliferation and motility

In our study, miR‐567 worked as an antagonist of cMras and regulated PTPRG expression. Intriguingly, PTPRG expression is also downregulated in lung cancer. Recent studies showed that PTPRG frequently functions as a tumour suppressor in tumour growth and development.^29^, ^30^ According to these evidences, our speculation was that miR‐567 and PTPRG might function as “tools,” controlled by regulatory molecules, such as circRNAs. Hence, in the present study, we demonstrated that cMras inhibited tumour growth and metastasis, working as a sponge of miRNA‐567 to directly restrain its activity, and subsequently upregulated its target PTPRG to exert its tumour suppressor function in LUAD (Figure 8).

## 5 CONCLUSION

Our work could be considered as an initial attempt in improving the knowledge regarding the role of cMras dysregulation in LUAD and its downstream molecular mechanisms through the miR‐567/PTPRG axis. Our findings provide novel evidences that circRNAs act as “microRNA sponges” and also provide a new therapeutic target and approach in the treatment of lung cancer.

## CONFLICT OF INTEREST

The authors declare no conflicts of interest.

## AUTHOR CONTRIBUTIONS

CY, FT, XZ and QW conceived and coordinated the study. CY, FT, JL, MS and MW carried out the experiments. CY and FT analysed the data. CY and FT wrote the manuscript. All authors have read and approved the final manuscript. CY, FT and JL were co‐first authors.
