# Supplementary figures and images for: Circular RNA cMras inhibits lung adenocarcinoma progression via modulating miR‐567/PTPRG regulatory pathway
Source: Cell Prolif. 2019 Apr 22;52(3):e12610. doi: 10.1111/cpr.12610 (PMC6536402; doi:10.1111/cpr.12610)

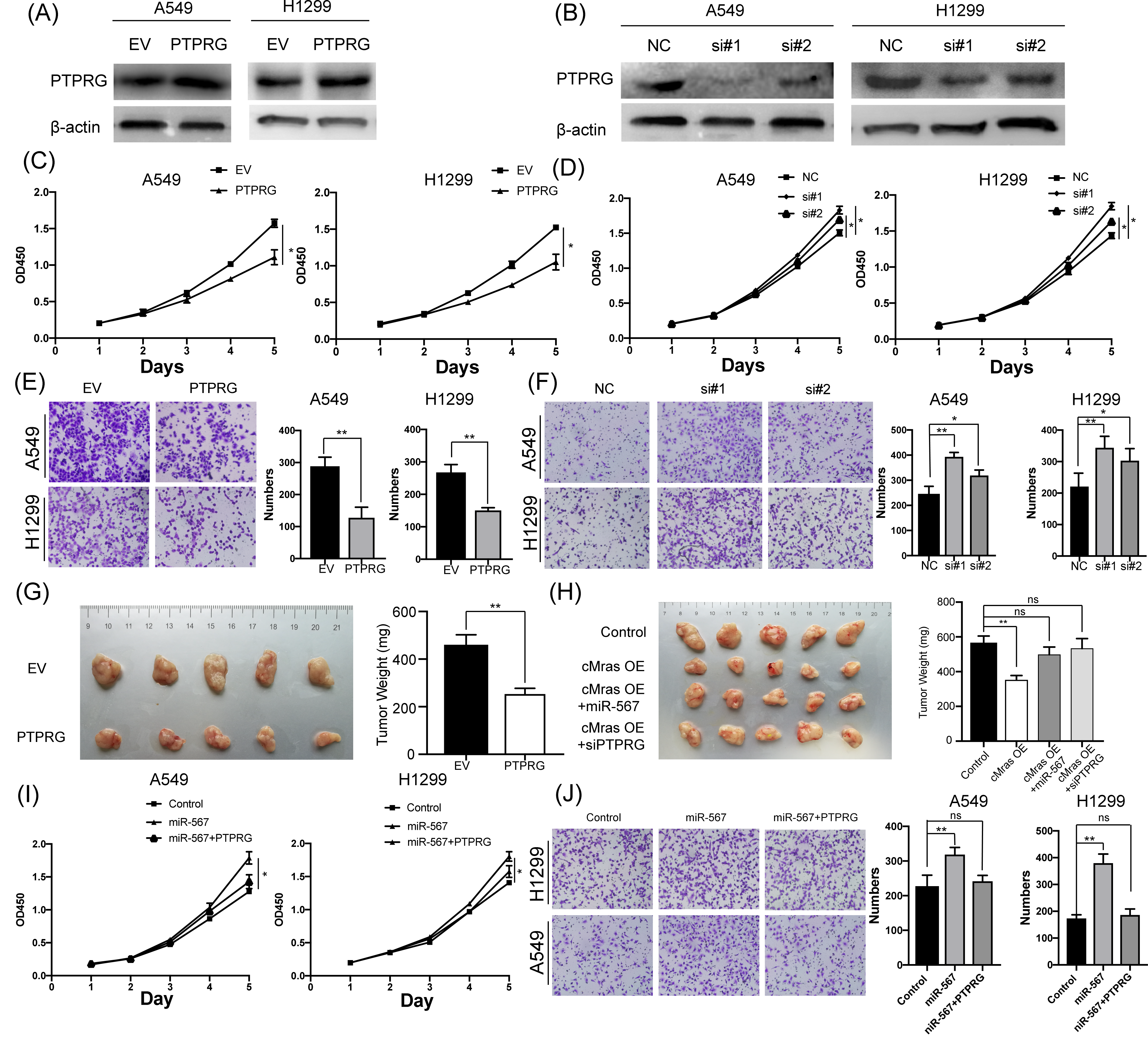

Supplement: Supplementary file 1 [file CPR-52-e12610-s001.tif]
