# Supplementary material for: Circular RNA cMras inhibits lung adenocarcinoma progression via modulating miR‐567/PTPRG regulatory pathway
Source: Cell Prolif. 2019 Apr 22;52(3):e12610. doi: 10.1111/cpr.12610 (PMC6536402; doi:10.1111/cpr.12610)
Supplement: Supplementary file 2 [file CPR-52-e12610-s002.docx]

**Supplemental_Table_1.** Primer sequences used in this study.

| ID | Primers |
| --- | --- |
| si-cMras#1 | CATCTTGGACGGTCTGACCTA |
| si-cMras#2 | TGGACGGTCTGACCTACGAGA |
| pzw-cMras-Forward | ﻿atagtagcaaattaaattcag |
| pzw-cMras-Reverse | ﻿ccctgattgctctggataggac |
| pcr-cMras-Forward | CAGCGCTCAATCCTTTGGG |
| pcr-cMras-Reverse | GACCTGCCACATTGGTCAGTA |
| pcr-U6-Forward | CTCGCTTCGGCAGCACA |
| pcr-U6-Reverse | AACGCTTCACGAATTTGCGT |
| pcr-GAPDH-Forward | ACTCCTCCACCTTTGACGC |
| pcr-GAPDH-Reverse | GCTGTAGCCAAATTCGTTGTC |
| pcr-PTPRG-Forward | GTATGGAGGCATGGCCAGTT |
| pcr-PTPRG-Reverse | CCGGAGGAAACGGAGGAAAA |
| pcr-EMP1-Forward | GCCAGTGAAGATGCCCTCAA |
| pcr-EMP1-Reverse | ATAGCCGTGGTGATACTGCG |
| pcr-DDX17-Forward | TCTGGAGTTGAGTGCCAACC |
| pcr-DDX17-Reverse | CGAGAACGACCACCCTTAC |
| luc-PTPRG -Forward | AAACTAGCGGCCGCTAGTTGACATTCCATGACGACATACATT |
| luc-PTPRG -Reverse | CTAGAATGTATGTCGTCATGGAATGTCAGCGGCCGCGTTT |
| luc-PTPRG mut Forward | AAACTAGCGGCCGCTAGTUGACAUUCCAUGACGTGTATGAT |
| luc-PTPRG mut -Reverse | CTAGATCATACACGTCATGGAATGTCAGCGGCCGCGTTT |
| luc-cMras-1 -Forward | AAAC TA GCGGCCGC TAGT tcctacctgaaacatacgT |
| luc-cMras-1 Reverse | CTAGAcgtatgtttcaggtaggaGCGGCCGCGTTT |
| luc-cMras-2 -Forward | AAAC TA GCGGCCGC TAGT aacctccccacatacaaT |
| luc-cMras-2 Reverse | CTAGA ttgtatgtggggaggtt GCGGCCGCGTTT |
| luc-cMras mut1 Forward | AAAC TA GCGGCCGC TAGT tcctacctgaTTGTATGAT |
| luc-cMras mut1 Reverse | CTAGATCATACAAtcaggtaggaGCGGCCGCGTTT |
| luc-cMras mut2 Forward | AAAC TA GCGGCCGC TAGTaacctcccctgtatgaT |
| luc-cMras mut2 Reverse | CTAGA tcatacaggggaggttGCGGCCGCGTTT |
| PTPRG-F(EcoR1) | ﻿atGAATTCatgcggaggttactggaaccg |
| PTPRG-R(EcoRv) | atGATATC﻿tcacactagggactccatgctc |
| si-PTPRG#1 | GGAGGTTTCCTGTTGAGAT |
| si-PTPRG#2 | GCTAATACCACTCGAATAT |
