# Supplementary material for: Circular RNA cMras inhibits lung adenocarcinoma progression via modulating miR‐567/PTPRG regulatory pathway
Source: Cell Prolif. 2019 Apr 22;52(3):e12610. doi: 10.1111/cpr.12610 (PMC6536402; doi:10.1111/cpr.12610)
Supplement: Supplementary file 3 [file CPR-52-e12610-s003.docx]

**Supplemental figure 1** (A) Western blot analysis of PTPRG in A549 and H1299 cells transfected with empty vector(EV), PTPRG overexpression vector.(B) Western blot analysis of PTPRG in A549 and H1299 cells transfected with negative control(NC), PTPRG siRNA#1 and PTPRG siRNA#2.(C, D) Effects of PTPRG on A549 and H1299 cell proliferation were measured by CCK-8 assays.(E, F) Representative images and bar graphs depicting the migration abilities of PTPRG overexpressing or PTPRG silenced in A549 and H1299 cells. (G) Effect of PTPRG on tumor growth in a nude mouse xenograft model. Representative images of tumors from the PTPRG and control groups (n = 5 for each group). (H) 2 × 10^6^ A549 cells transfected with control vector or PTPRG overexpression vector were inoculated subcutaneously into armpit of nude mice, all of the mice examined developed tumors at 10th day. miR-567 mimics or siRNA against PTPRG(PTPRG siRNA#1) were injected in cMras overexpressing tumors for the next 20 days. cMras derived tumors showed smaller tumor weight compared with the control tumors. (I) Growth curves for A549 and H1299 cells after co-transfection with control, miR-567 mimics, or the combination of miR-567 and PTPRG were determined by CCK8. (J) Migration ability for A549 and H1299 cells after co-transfection with control, miR-567 mimics, or the combination of miR-567 and PTPRG were determined by transwell assay. *P* < 0.05 *, *P* < 0.01 **.
